# Food induced anaphylaxis in Irish children

**DOI:** 10.1186/2045-7022-5-S3-P102

**Published:** 2015-03-30

**Authors:** Ioana Maris, Ronan O'Sullivan, Jonathan Hourihane

**Affiliations:** 1 Cork University Hospital, Cork, Ireland

## Background

Recent studies suggest that the incidence of anaphylaxis is increasing, particularly among young children. Food allergens represent the most frequent triggers in children.

## Aim

To determine the key features of anaphylactic reactions and their emergency treatment in children presenting with anaphylaxis to the Paediatric Units or associated Emergency Departments in Ireland.

## Method

An IPSU monthly multi-study report card is sent to every paediatrician, and anaphylaxis is included in this card from September 2013 to March 2015. Data are gathered using EAACI's Anaphylaxis Registry Form.

## Results

51 reported anaphylaxis cases to date, with data available for 37. There were 23 (62%) boys and 27 (73%) new patients in this group. Food was the implicated allergen in 29/37 (78%). Peanut and cashew were the most frequent foods involved (24%), followed by egg (17%) and milk (14%). 62% of the cases were exposed to food allergens at home. Skin (92%) and airway symptoms (89%) predominated, with gastro-intestinal (48%) and cardio-vascular symptoms (46%) also frequent. One fatal anaphylaxis occurred, due to peanut. 84% of cases presented to hospital either directly (51%) or referred by a General Practitioner (32%). Adrenaline i.m. was given in 21/37 cases (57%). Adrenaline was self/parent-injected in 4 cases (19%), by a GP in 10 (47%), after arrival in hospital in 6 (28%), and by paramedics in 1 case. In the 10 known patients group, 3/6 (50%) used their prescribed Adrenaline Auto injectors, and 4/10 (40%) had not been prescribed Adrenaline.

## Conclusion

Food allergens are the main triggers for anaphylaxis in children. The rate of Adrenaline usage clearly needs to improve in Ireland and low usage of prescribed Adrenaline emphasizes the need for educational and other support strategies for patients and physicians.

